# Preoperative anxiety and depression symptoms are associated with poorer clinical outcomes following corrective surgery for adult equinocavovarus foot

**DOI:** 10.3389/fpsyt.2026.1803760

**Published:** 2026-04-15

**Authors:** JunKui Xu, Dong Hu, Jun Lu, Yi Li, ZiRui Yu

**Affiliations:** Honghui Hospital, Xi’an Jiaotong University, Xi’an, Shanxi, China

**Keywords:** adult, anxiety, corrective surgery, correlation, depression, equinocavovarus foot, prognosis

## Abstract

**Purpose:**

This study aimed to investigate the preoperative psychological status of adult patients with equinocavovarus foot deformity and to examine the association between preoperative anxiety/depressive symptoms and the clinical outcomes of corrective surgery in this population.

**Methods:**

A retrospective analysis was conducted on 103 adult patients who underwent corrective surgery for equinocavovarus foot at Xi’an Honghui Hospital between March 2014 and July 2023. Baseline data were collected. Patient psychological status, ankle-hindfoot function, pain, and quality of life were assessed preoperatively and at the final follow-up using the Hospital Anxiety and Depression Scale (HADS), the American Orthopedic Foot & Ankle Society (AOFAS) ankle-hindfoot score, the Visual Analog Scale (VAS), and the 36-Item Short Form Health Survey (SF-36). Based on preoperative HADS scores, patients were categorized into an anxiety/depression group (Group A) and a non-anxiety/depression group (Group B). The two groups were compared with respect to baseline characteristics (gender, age, disease duration, BMI, follow-up duration), clinical outcomes, and the degree of improvement in all assessment metrics.

**Results:**

A total of 83 patients completed the follow-up, among whom 38 (45.78%) exhibited preoperative anxiety/depression symptoms. No significant differences were found in baseline characteristics between the two groups (all P > 0.05). At the final follow-up, both groups showed significant improvement in VAS, AOFAS, SF-36 (PCS/MCS), and HADS (A/D) scores compared to their preoperative baselines (all P < 0.001). Intergroup comparisons revealed that Group A had significantly lower AOFAS and SF-36 (PCS/MCS) scores, and significantly higher VAS and HADS (A/D) scores than Group B, both preoperatively and at the final follow-up (all P < 0.001). Regarding the degree of improvement, Group A demonstrated a smaller magnitude of improvement in VAS (P < 0.01), AOFAS (P < 0.01), and SF-36 PCS (P < 0.001) compared to Group B. Conversely, Group A showed a greater improvement in SF-36 MCS and HADS (A/D) scores (all P < 0.001).

**Conclusions:**

While surgery improved all outcomes, patients with preoperative anxiety/depression exhibited persistently worse clinical scores. Their improvement profile was distinct: smaller gains in pain and physical function but greater mental health improvement. Addressing preoperative psychological status may optimize comprehensive outcomes.

## Highlights

- Studies investigating the psychological health of adults with equinocavovarus foot deformity.
- 46% prevalence of preoperative anxiety/depression in this surgical population.
- Preoperative anxiety/depression is significantly associated with poorer clinical outcomes following corrective surgery.

## Introduction

The equinocavovarus foot is a complex deformity characterized by varying degrees of hindfoot varus, equinus, and pes cavus, accompanied by forefoot adduction and supination (1–3). Although the condition is primarily treated during childhood, approximately 33-85% of patients in developing countries experience delayed treatment for various reasons, leading to the persistence of the deformity into adulthood, a condition termed adult equinocavovarus foot (3–6). The etiology of adult equinocavovarus foot is complex and multifactorial. In addition to neglected congenital equinocavovarus foot, it encompasses various secondary causes, including sequelae of neuromuscular diseases (e.g., poliomyelitis, spina bifida), post-traumatic deformities, and infectious diseases (2, 7). In contrast to pediatric cases, adult patients often present with severe bony deformities, joint degeneration, and soft tissue contractures. These pathological changes severely impact the patient’s quality of life and social functioning through persistent pain, abnormal gait, and difficulty with footwear (7, 8). Given the maturity of adult skeletal development, deformity fixation, and the presence of secondary degenerative changes, conservative treatment has limited effectiveness, and surgical correction has become the main treatment strategy for restoring foot function and improving quality of life (3, 9–11).

In recent years, a variety of surgical techniques have been applied in the management of adult equinocavovarus foot. Gursu et al. treated severe adult equinocavovarus foot deformities with talectomy and tibiocalcaneal arthrodesis with intramedullary nailing fixation, which improved the mean AOFAS score from 41.1 to 78.4, and the mean VAS score from 6.3 to 0.8 postoperatively (9). Du et al. found that the application of minimally invasive U-osteotomy combined with a Taylor Spatial Frame for gradual correction is an effective and relatively safe method for managing severe rigid equinocavovarus foot deformities in adults (12).However, despite continuous improvements in surgical techniques, approximately 25% of patients still report dissatisfaction with their postoperative outcomes (13, 14). More and more evidence indicates that, apart from surgical technical factors, a patient’s mental health status may be a significant factor influencing the prognosis of surgery. Nakagawa et al. reported that approximately 30% of patients with chronic foot and ankle conditions exhibited symptoms of anxiety or depression (15). Among patients undergoing hallux valgus surgery, Goh et al. found that those with preoperative emotional disorders demonstrated poorer VAS, AOFAS, and SF-36 (MCS) scores at baseline and 6-month after surgery, compared to those without such disorders (16). Similar findings have been corroborated in other Orthopedic surgeries, where preoperative anxiety and depression have been associated with persistent postoperative pain, suboptimal functional recovery, and an increased risk of complications (17–19). Collectively, these studies imply that preoperative mental health assessment and intervention may hold significant potential for improving surgical prognosis in the management of adult equinocavovarus foot.

Currently, research on surgical correction for adult equinocavovarus foot mainly focuses on the refinement of surgical techniques and the optimization of deformity correction strategies, while there is a significant lack of attention to non-technical factors affecting surgical prognosis, particularly the patient’s psychological status. Existing studies have indicated that the mental health outcomes reported by patients with lower limb deformities accompanied by Angle deformities are significantly worse than those of the control group (20). Therefore, it is necessary to direct clinical attention to the psychological well-being of this patient population. Given the established correlation between psychological factors and postoperative prognosis in other orthopedic surgeries, exploring the role of this factor in adult equinocavovarus foot surgery holds significant clinical relevance. The present study aimed to clarify the correlation between psychological factors and surgical outcomes by conducting preoperative psychological assessments and comparing clinical outcomes between patients with and without preoperative anxiety/depressive symptoms. The findings of this study are expected to provide a scientific basis for establishing a comprehensive preoperative assessment system that incorporates mental health evaluation, identifying high-risk patient groups, and formulating integrated psychological-surgical treatment protocols.

## Patients and methods

### Patients

This study was approved by the Institutional Review Board (Approval No: 2025-KY-175-01). A retrospective analysis was conducted on patients who underwent corrective surgery for equinocavovarus foot deformity in the Comprehensive Foot and Ankle Ward of Xi’an Honghui Hospital between March 2014 and July 2023. Informed consent was obtained from all participants. The inclusion criteria were as follows: (1) equinocavovarus foot corrected by surgery; (2) at least 18 years old; (3) capable of completing the questionnaire survey independently. The exclusion criteria were as follows: (1) a history of traumatic surgery on the affected limb; (2) diagnosis of cerebral palsy; (3) bedridden status with an inability to walk; (4) presence of severe osteoporosis, rheumatoid arthritis, other arthritic conditions, or ankle joint infection; (5) presence of chronic medical diseases such as diabetes, hypertension, malignant tumors, or hepatic/renal insufficiency; (6) a history of treatment for mental disorders.

### Methods

Baseline data, including patient’s gender, age, body mass index (BMI), and disease duration, were collected through the hospital’s case system. Assessments were conducted preoperatively and at the final follow-up using the Hospital Anxiety and Depression Scale (HADS), the American Orthopedic Foot & Ankle Society (AOFAS) ankle-hindfoot score, the Visual Analog Scale (VAS) for pain, and the 36-Item Short Form Health Survey (SF-36) to evaluate mental health status, functional activity, pain, and quality of life, respectively.

The HADS consists of two subscales: anxiety (HADS-A) and depression (HADS-D). Each subscale comprises 7 items designed to assess the severity of anxiety or depression. Each item is scored from 0 to 3, with a total subscale score ranging from 0 to 21. A critical value of ≥8 on either subscale indicates the presence of clinically significant anxiety or depressive symptoms (21). The VAS consists of a 100-mm horizontal line (22). The left endpoint, labeled “0” represents “no pain,” while the right endpoint, labeled “100” represents “the most intense pain imaginable.” Participants are instructed to mark the line to indicate their current pain level. The score is determined by measuring the distance in millimeters from the left endpoint to the mark, resulting in a range from 0 to 100. A higher score indicates more severe pain symptoms (22). The AOFAS scoring system has a maximum of 100 points. It assesses the comprehensive function of the ankle and hindfoot through a subjective pain component (40 points) and objective components for function (50 points) and alignment (10 points). Currently, the system has been widely applied in functional evaluation before and after surgery for ankle and hindfoot disorders. For corrective surgery of equinocavovarus foot deformity, the AOFAS score ranges from 0 to 100, with a higher score indicating better ankle-hindfoot function (23). The SF-36 consists of eight subscales which are aggregated into two summary measures, with the first four being the Physical Component Summary (PCS) and the last four being the Mental Component Summary (MCS) (24). It has been widely used as an indicator to measure physical and mental dysfunction in the Chinese population (25). The scores for both the SF-36 (PCS) and SF-36 (MCS) range from 0 to 400. A higher score indicates a better health status. In this study, patients with a preoperative HADS-A or HADS-D subscale score of ≥8 were assigned to the group with anxiety/depression symptoms (Group A), and the remaining patients were assigned to the group without these symptoms (Group B).

**Figure 1:**
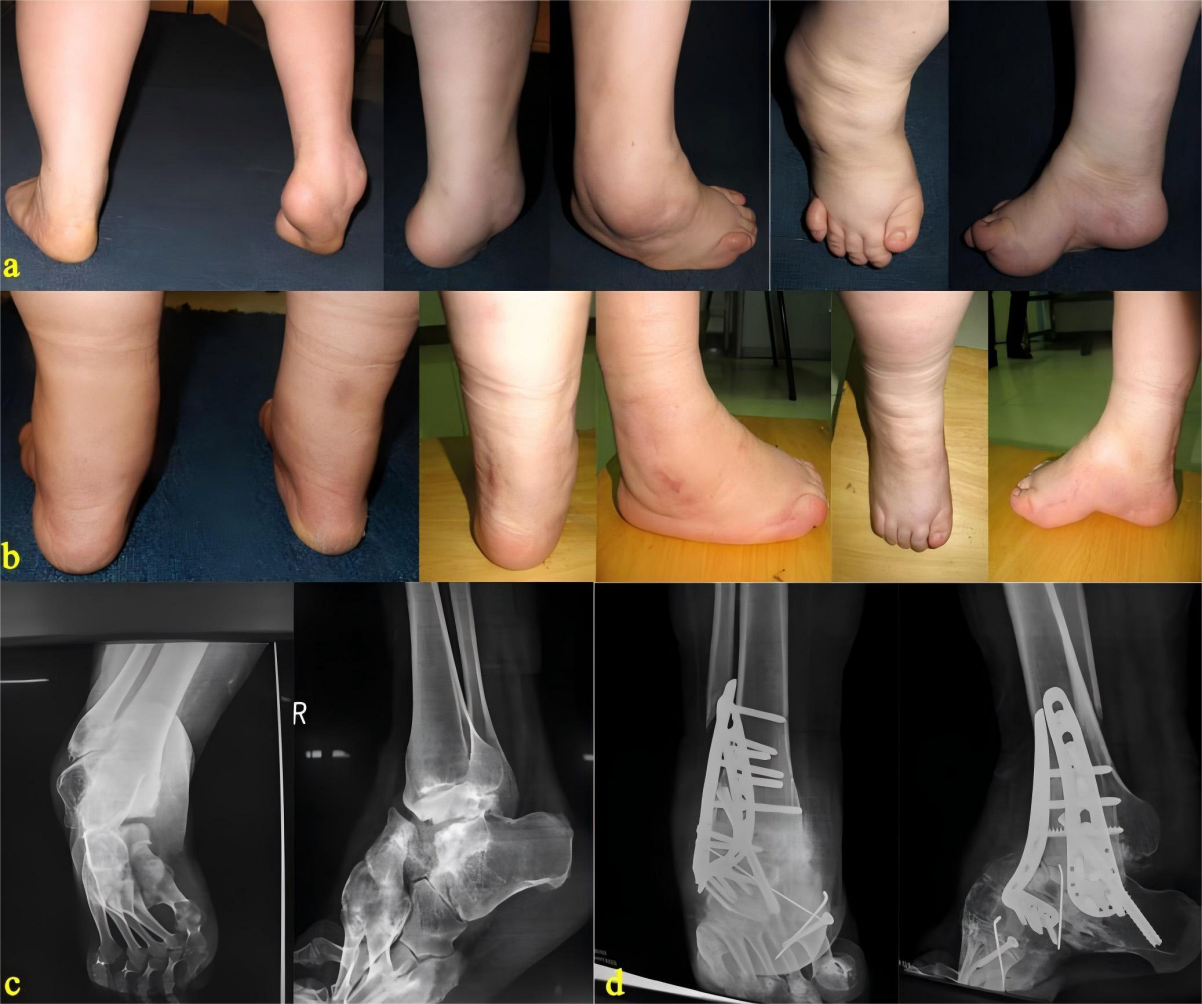
Changes in the patient's appearance and X-ray findings preoperative and postoperative. **(a)** Preoperative appearance of the foot, showing hindfoot varus, a high arch, and a talipes equinus deformity; **(b)** Postoperative appearance of the foot, demonstrating significant improvement in hindfoot alignment and the high-arch deformity; **(c)** Preoperative X-ray, revealing marked degeneration in the midfoot and hindfoot joints, with most foot joints exhibiting malalignment; **(d)** Postoperative X-ray, showing extensive joint corrective fusion performed, with the deformity well corrected.

Although the surgical techniques were not strictly standardized, all procedures were performed under general anesthesia combined with peripheral nerve blocks and were conducted by the same team of experienced senior consultant foot and ankle surgeons. This ensured relative consistency in surgical philosophy and technical details. Postoperatively, all patients received standardized rehabilitation guidance to guarantee a uniform recovery process, thereby facilitating a fair comparison of surgical outcomes. **Figure 1** show the preoperative and postoperative appearance and radiographic images of a patient’s right foot.

### Statistical analysis

Statistical analysis was performed on all data using SPSS software (version 27.0; IBM, New York, USA). The normality of continuous variables was assessed using the Shapiro-Wilk test. The results indicated that the data deviated from a normal distribution; consequently, non-parametric methods were adopted for subsequent analyses. For descriptive statistics, continuous data were expressed as the median with interquartile range (IQR), presented in the format M (P25, P75). For continuous variables, within-group comparisons for paired samples were performed using the Wilcoxon signed-rank test, while between-group comparisons were conducted using the Mann–Whitney U test. Effect sizes are reported as the Hodges–Lehmann median difference (Med-diff) with its 95% confidence interval (CI), along with the rank-biserial correlation (r) as an estimate of the magnitude of the observed effects. Categorical data are presented as frequency (n) and percentage (%). Differences between groups for binary outcomes such as sex were assessed using the Chi-square test, with risk difference (RD) and odds ratio (OR) reported alongside their 95% CIs. A two-sided significance level of 0.05 was used for all statistical tests, with P < 0.05 considered statistically significant.

## Results

### General characteristics of the patients

A total of 103 eligible patients were included in this study, among whom 83 patients received complete follow-up, including 44 males and 39 females. The median age of the patients was 33.00 years (IQR: 25.00, 46.00), with a median disease duration of 156.00 months (IQR: 60.00, 360.00). The median BMI was 23.53 (IQR: 21.77, 25.39), and the median follow-up duration was 43.00 months (IQR: 31.00, 66.00). Patients were divided into two groups according to the preoperative HADS score. Group A comprised 38 patients (accounting for 45.78%), including 20 males and 18 females. Group B comprised 45 patients (accounting for 54.22%), including 24 males and 21 females. No statistically significant differences were observed in baseline characteristics between the two groups (all P > 0.05), The effect sizes for all variables were negligible (r: from -0.19 to 0.11; odds ratio for sex: 0.01 [95% CI: 0.00 to 0.22]), indicating that the magnitude of the between-group differences was clinically insignificant. The baseline characteristics of both groups are presented in **Table 1**.

**Table 1:** Baseline characteristics of patients in group A and group B.

Variables	Group A (n=38)	Group B (n=45)	P-value	Group difference (95% CI)	Effect size (95% CI)
Sex (Male)	20 (52.6%)	24 (53.3%)	0.95	RD: -0.7% (-22.3%, 20.9%)	OR: 0.01 (0.00, 0.22)
Age (years)	33.50 (26.50, 44.00)	32.00 (25.00, 48.50)	0.94	Med diff: 0.00 (-7.00, 5.00)	r: 0.01 (-0.21, 0.23)
Disease duration (months)	126.00 (60.00, 360.00)	204.00 (60.00, 438.00)	0.41	Med diff: -24.00 (-120.00, 41.00)	r: -0.09 (-0.31, 0.13)
BMI (kg/m^2^ )	22.75 (21.57, 24.29)	24.80 (21.87, 25.67)	0.08	Med diff: -1.37 (-2.53, 0.20)	r: -0.19 (-0.41, 0.02)
Follow-up duration (Month)	46.00 (30.50, 73.50)	42.00 (30.50, 59.00)	0.34	Med diff: 4.00 (-4.00, 14.00)	r: 0.11 (-0.11, 0.32)

Group A, Patients with preoperative anxiety/depression symptoms; Group B, Patients without preoperative anxiety/depression symptoms.

RD, Risk Difference; OR, Odds Ratio; Med diff, Median difference (Hodges-Lehmann estimator); r, Rank-biserial correlation.

Data presentation: n (%) for Sex, median (IQR) represents other indicators.

### Clinical evaluation of corrective surgery

**Figure 2:**
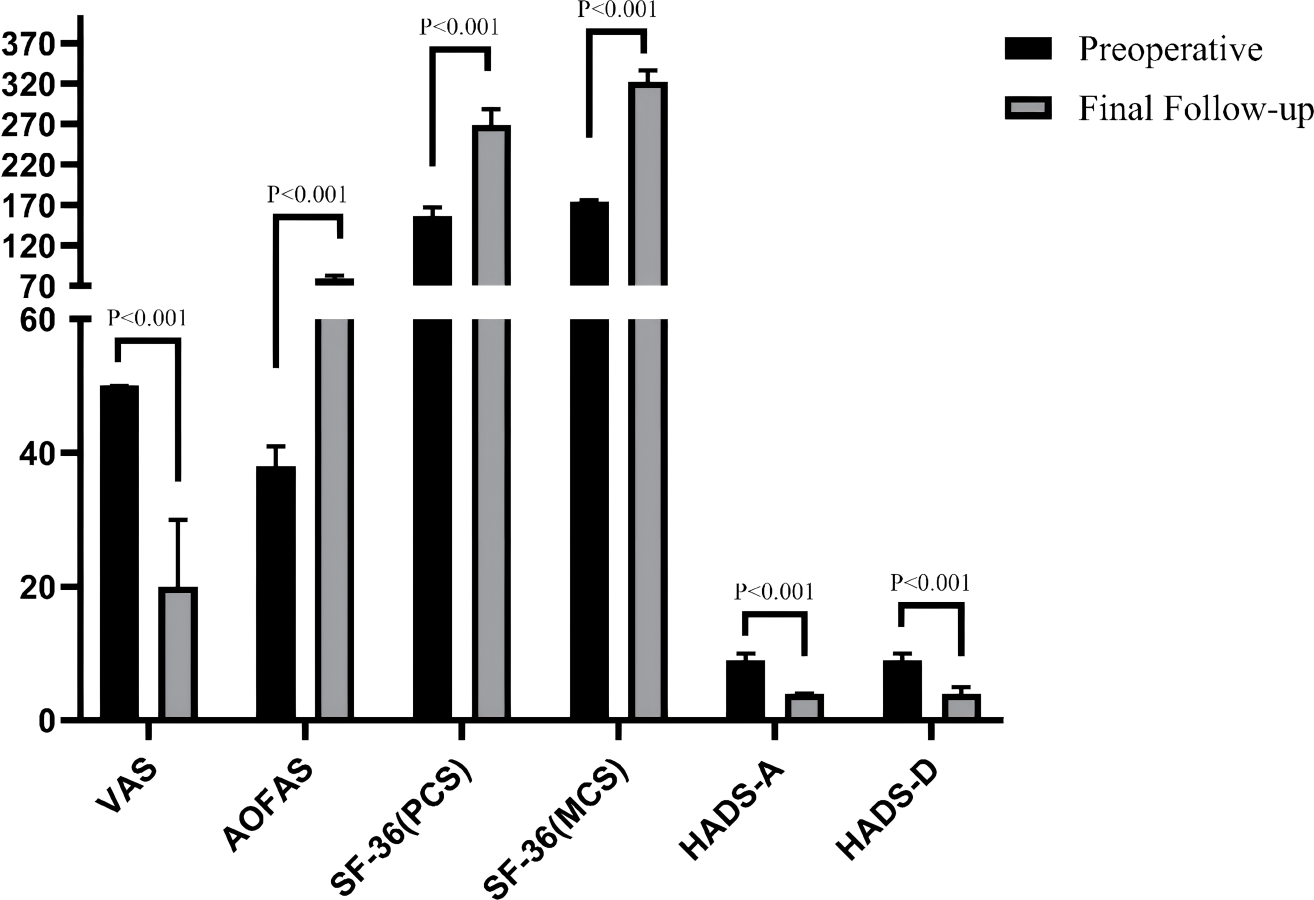
Comparison of clinical evaluation indicators between preoperative and final follow-up in group A patients.

**Figure 3:**
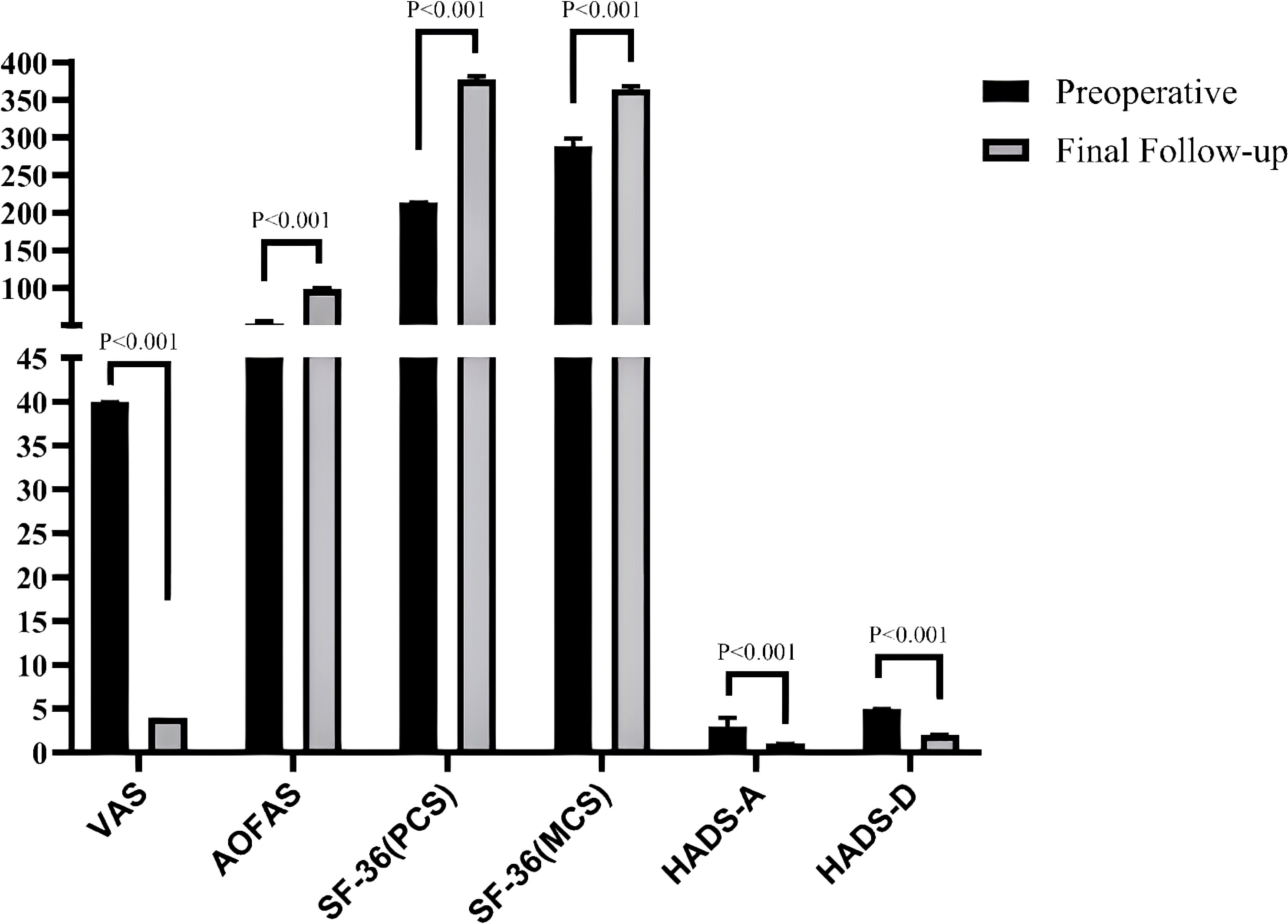
Comparison of clinical evaluation indicators between preoperative and final follow-up in group B patients.

A comparison results within each group (preoperative vs. final follow-up) of the VAS, AOFAS, SF-36 (PCS), SF-36 (MCS), HADS-A, and HADS-D revealed that all outcome measures in both groups were significantly improved at the final follow-up compared to preoperative assessments (all P < 0.001), The effect sizes were consistently large (r: from -0.87 to -0.89 in Group A and from -0.79 to -0.87 in Group B), indicating that the corrective surgery yielded robust and clinically meaningful effects, as shown in **Tables 2**, **3**, **Figures 2**, **3**.

**Table 2:** Comparative analysis of clinical outcome measures in group A: preoperative vs. final follow-up.

Variables	Paired [M(P25, P75)]		p-value	Median difference (95% CI)	Effect size (r) (95% CI)
	Preoperative	Final follow-up			
VAS	50.00 (40.00, 50.00)	20.00 (13.75, 30.00)	<0.001	-27.50 (-30.50, -25.00)	-0.87 (-1.00, -0.55)
AOFAS	38.00(34.75, 41.00)	79.00 (72.00, 83.00)	<0.001	40.00 (37.50, 42.50)	-0.87 (-1.00, -0.56)
SF-36(PCS)	156.00 (146.00,167.00)	269.00 (243.25, 288.00)	<0.001	107.50 (94.00, 121.50)	-0.87 (-1.00, -0.55)
SF-36(MCS)	174.00 (172.00, 176.00)	322.00 (310.00, 336.00)	<0.001	143.50 (133.00, 152.50)	-0.87 (-1.00, -0.56)
HADS-A	9.00 (8.00, 10.00)	4.0 0 (3.00, 4.00)	<0.001	-5.50 (-5.50, -5.00)	-0.89 (-1.00, -0.57)
HADS-D	9.00 (8.00, 10.00)	4.00 (4.00, 5.00)	<0.001	-5.00 (-5.00, -4.00)	-0.89(-1.00, -0.57)

VAS, Visual Analog Scale; AOFAS, American Orthopedic Foot & Ankle Society; SF-36 (PCS), 36-Item Short Form Health Survey (Physical component summary); SF-36 (MCS), 36-Item Short Form Health Survey (Mental component summary); HADS-A, Hospital anxiety and depression scale – Anxiety; HADS-D, Hospital anxiety and depression scale - Depression.

**Table 3:** Comparative analysis of clinical outcome measures in group B: preoperative vs. final follow-up.

Variables	Paired [M (P25, P75)]		p-value	Median difference (95% CI)	Effect size (r) (95% CI)
	Preoperative	Final follow-up			
VAS	40.00 (32.50, 40.00)	4.00 (4.00, 4.00)	<0.001	-31.00 (-33.50, -30.50)	-0.79 (-1.00, -0.50)
AOFAS	52.00 (49.00, 55.50)	98.00 (91.50, 100.00)	<0.001	44.50 (42.00, 46.00)	-0.80 (-1.00, -0.51)
SF-36 (PCS)	214.00 (203.50, 214.00)	377.00 (352.00, 382.00)	<0.001	158.00 (151.50, 163.00)	-0.87 (-1.00, -0.58)
SF-36 (MCS)	289.00 (274.50, 298.50)	364.00 (364.00, 368.00)	<0.001	76.50 (71.00, 84.00)	-0.87 (-1.00, -0.58)
HADS-A	3.00 (2.00, 4.00)	1.00 (0.00, 1.00)	<0.001	-2.50 (-2.50, -2.00)	-0.82 (-1.00, -0.52)
HADS-D	5.00 (4.00, 5.00)	2.00 (1.00, 2.00)	<0.001	-3.00 (-3.00, -2.50)	-0.81 (-1.00, -0.52)

### Correlation analysis of preoperative mental health status with clinical outcome measures and surgical prognosis

**Figure 4:**
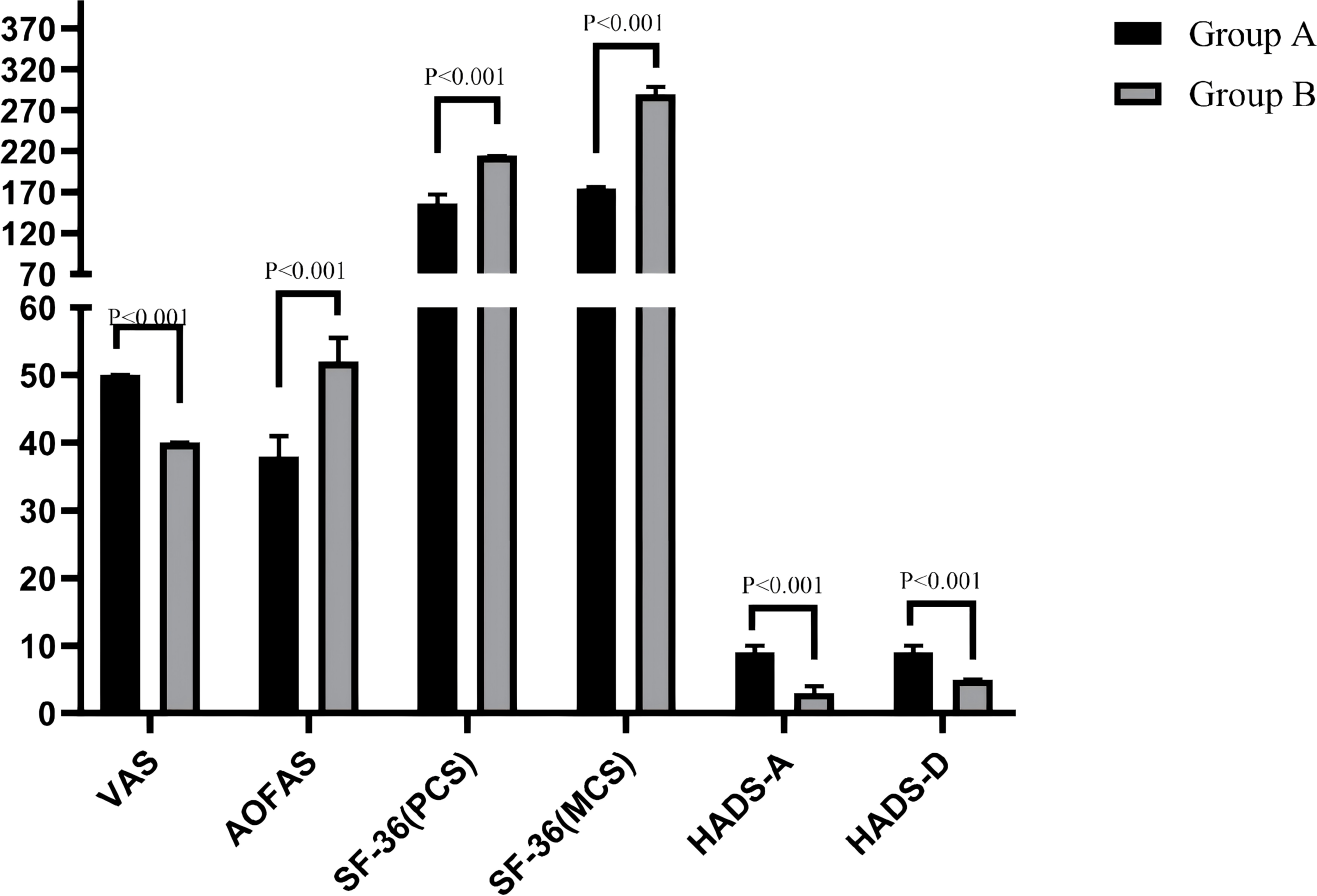
Comparison of preoperative clinical evaluation indicators between groups.

**Figure 5:**
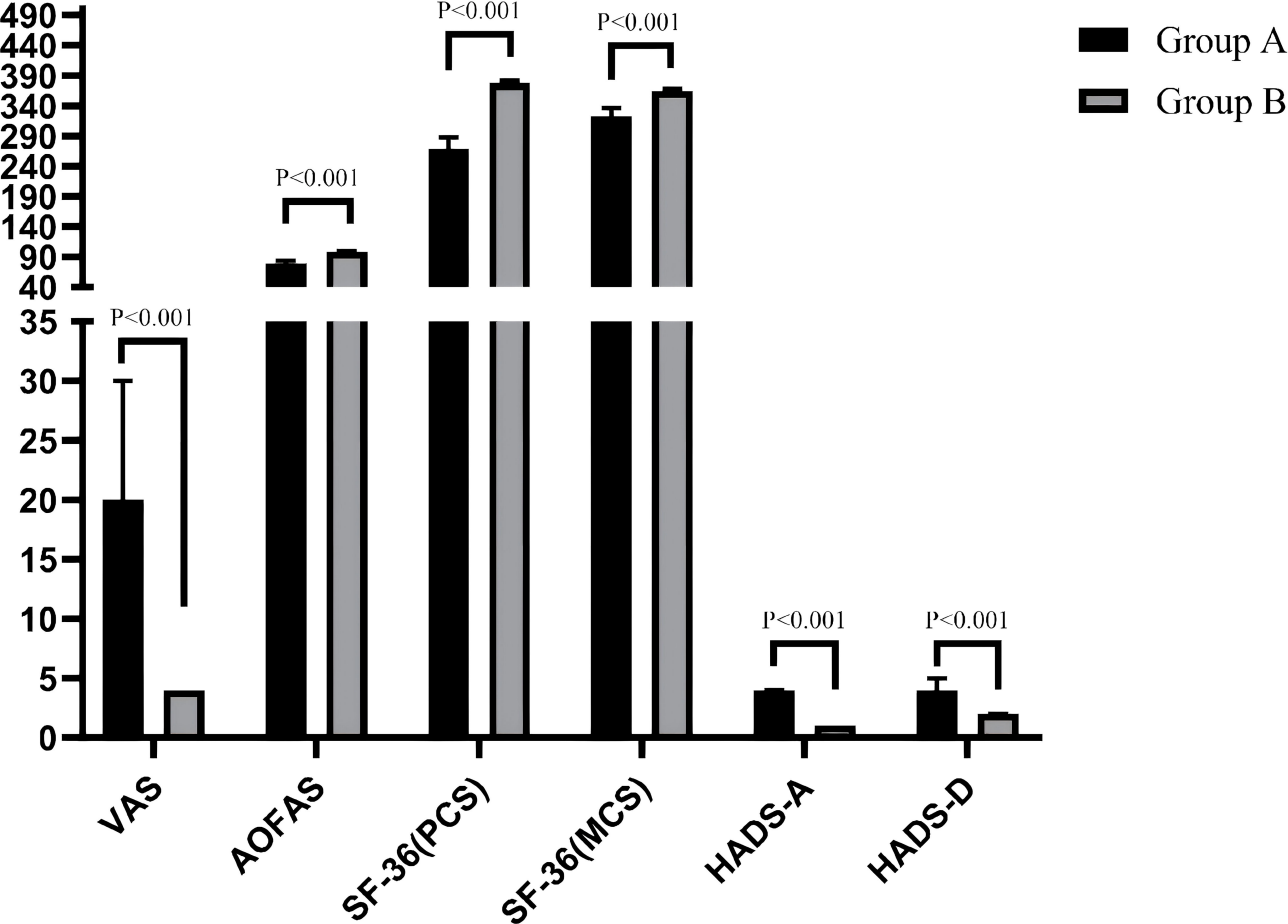
Comparison of clinical evaluation indicators at the final follow-up between groups.

This study compared the VAS, AOFAS, SF-36 (PCS), SF-36 (MCS), HADS-A, and HADS-D scores between the two groups both preoperatively and at the final follow-up. The results demonstrated that scores for all outcome measures in Group A were significantly worse than those in Group B at both time points (all P < 0.001), The effect sizes ranged from moderate to very large (preoperatively: r: from 0.58 to 0.87; postoperatively: r: from 0.65 to 0.85), indicating clinically meaningful differences between the two groups at both baseline and final follow-up, as shown in **Tables 4**, **5**, **Figures 4**, **5**.

**Table 4:** Analysis of differences in preoperative clinical outcome measures between groups.

Variables	Paired [M (P25, P75)]		p-value	Median difference (95% CI)	Effect size (r) (95% CI)
	Group A (n=38)	Group B (n=45)			
VAS	50.00 (40.00, 50.00)	40.00 (32.50, 40.00)	<0.001	10.00 (10.00, 10.00)	0.58 (0.37, 0.80)
AOFAS	38.00 (34.75, 41.00)	52.00 (49.00, 55.50)	<0.001	-14.00 (-17.00, -12.00)	-0.77 (-0.99, -0.56)
SF-36 (PCS)	156.00 (146.00, 167.00)	214.00 (203.50, 214.00)	<0.001	-55.00 (-63.00, -47.00)	-0.85 (-1.00, -0.63)
SF-36 (MCS)	174.00 (172.00, 176.00)	289.00 (274.50, 298.50)	<0.001	-113.00 (-121.00, -108.00)	-0.86 (-1.00, -0.65)
HADS-A	9.00 (8.00, 10.00)	3.00 (2.00, 4.00)	<0.001	6.00 (5.00, 6.00)	0.87 (0.65, 1.00)
HADS-D	9.00 (8.00,10.00)	5.00 (4.00, 5.00)	<0.001	4.00 (4.00, 5.00)	0.87 (0.65, 1.00)

**Table 5:** Analysis of differences in clinical outcome measures between groups at final follow-up.

Variables	Paired [M (P25, P75)]		p-value	Median difference (95% CI)	Effect size (r) (95% CI)
	Group A (n=38)	Group B (n=45)			
VAS	20.00 (13.75, 30.00)	4.00 (4.00, 4.00)	<0.001	16.00 (11.00, 16.00)	0.65 (0.44, 0.87)
AOFAS	79.00 (72.00, 83.00)	98.00 (91.50, 100.00)	<0.001	-18.00 (-21.00, -15.00)	-0.81 (-1.00, -0.60)
SF-36 (PCS)	269.00 (243.25, 288.00)	377.00 (352.00, 382.00)	<0.001	-101.00 (-115.00, -90.00)	-0.82 (-1.00, -0.61)
SF-36 (MCS)	322.00 (310.00, 336.00)	364.00 (364.00, 368.00)	<0.001	-42.00 (-47.00, -36.00)	-0.83 (-1.00, -0.62)
HADS-A	4.00 (3.00, 4.00)	1.00 (0.00, 1.00)	<0.001	3.00 (3.00,3.00)	0.85 (0.63, 1.00)
HADS-D	4.00 (4.00, 5.00)	2.00 (1.00, 2.00)	<0.001	2.00 (2.00, 3.00)	0.83 (0.62, 1.00)

**Figure 6:**
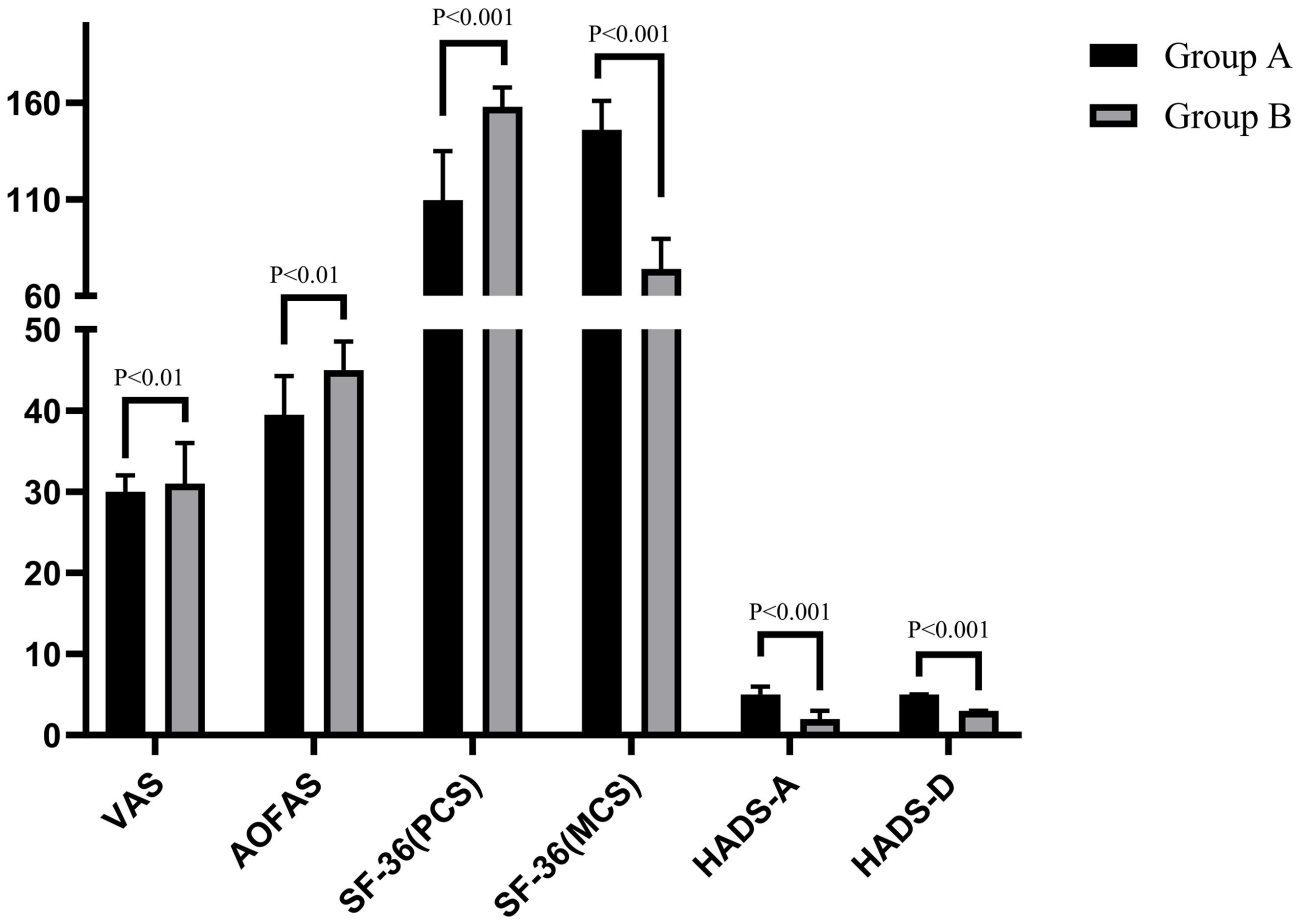
Comparison of the improvement various indicators from preoperative to final follow-up between groups.

A further intergroup analysis was conducted to compare the improvement degrees (from preoperative to final follow-up) of VAS, AOFAS, SF-36 (PCS), SF-36 (MCS), HADS-A, and HADS-D scores. The results indicated that the improvement degrees of SF-36 (MCS), HADS-A, and HADS-D scores were significantly greater in Group A than in Group B (all P < 0.001, r: from 0.75 to 0.85). In contrast, the improvement degrees of VAS (P < 0.01, r: -0.28 [95% CI: -0.50 to -0.07]), AOFAS (P < 0.01, r: -0.31 [95% CI: -0.53 to -0.10]), and SF-36 (PCS) (P < 0.001, r: -0.69 [95% CI: -0.82 to -0.39]) scores were significantly lower in Group A compared to Group B. The patterns of improvement across different domains differed significantly between the groups. For detailed results, please refer to **Table 6**, **Figure 6**.

**Table 6:** Comparative analysis of the magnitude of improvement in outcome measures from preoperative to final follow-up between groups.

Variables	Paired [M (P25, P75)]		p-value	Median difference (95% CI)	Effect size (r) (95% CI)
	Group A (n=38)	Group B (n=45)			
VAS	30.00 (20.00, 32.00)	31.00 (26.00, 36.00)	<0.01	-5.00 (-6.00, 0.00)	-0.28 (-0.50, -0.07)
AOFAS	39.50 (36.00, 44.25)	45.00 (39.00, 48.50)	<0.01	-4.00 (-7.00, -1.00)	-0.31 (-0.53, -0.10)
SF-3 (PCS)	109.50 (79.00, 135.00)	158.00 (143.00, 168.00)	<0.001	-48.00 (-62.00, -34.00)	-0.60 (-0.82, -0.39)
SF-36(MCS)	146.00 (121.50, 161.00)	74.00 (64.50, 89.50)	<0.001	67.00 (55.00, 78.00)	0.75 (0.53, 0.97)
HADS-A	5.00 (5.00, 6.00)	2.00 (2.00, 3.00)	<0.001	3.00 (3.00, 3.00)	0.85 (0.64, 1.00)
HADS-D	5.00 (4.00, 5.00)	3.00 (2.50, 3.00)	<0.001	2.00 (2.00, 2.00)	0.79 (0.57, 1.00)

## Discussion

To the best of our knowledge, no study has specifically investigated the association between preoperative mental health status and surgical prognosis in patients with adult equinocavovarus foot deformity. The present study provides the first systematic evaluation of this relationship. Currently, corrective intervention for equinocavovarus foot deformity is predominantly performed during childhood. Consequently, research specifically targeting adult patients remains relatively limited (26), and existing studies mainly focus on the refinement and optimization of surgical techniques. Although combined surgical techniques have achieved satisfactory clinical outcomes in the correction of adult equinocavovarus foot deformity (3, 9, 12, 13), these studies generally overlooked the potential influence of patients’ mental health factors on treatment outcomes. In light of this, the present study conducted preoperative mental health screening using standardized scales in adults with equinocavovarus foot deformity and systematically analyzed the correlation between preoperative anxiety/depression symptoms and clinical manifestations and surgical outcomes, aiming to provide evidence-based medical evidence for the formulation of comprehensive treatment strategies for this patient population.

The results of this study demonstrated that all patients, regardless of the presence or absence of preoperative anxiety/depression symptoms, showed significant improvements in all scores at the final follow-up compared to preoperative assessments (all P < 0.001). Equinocavovarus foot deformity typically causes pain, abnormal gait, and non-plantigrade foot posture in patients (7, 8), resulting in poorer preoperative clinical scores. After corrective surgery, patient’s symptoms were significantly alleviated, enabling most to resume normal work and daily activities (9, 10). Furthermore, studies have found that reduced postoperative pain and improved functional capacity can lead to a significant amelioration of anxiety and depression symptoms (27). Multiple studies on corrective surgery for adult equinocavovarus foot deformity have demonstrated significant improvements in the AOFAS score (3, 9, 12, 13, 28, 29) and VAS score (9, 29) at the final follow-up compared to preoperative levels, along with the achievement of a plantigrade foot (3, 9, 12–14, 29). More importantly, the positive impact of surgery on mental health and quality of life extends to other foot and ankle conditions, such as hallux valgus (30), end-stage ankle arthritis (31), and painful accessory navicular syndrome (32). These cross-disease consistent findings suggest that the value of corrective surgery of foot and ankle pathologies extends beyond anatomical reconstruction. Its greater significance lies in facilitating a holistic enhancement of patient well-being, both physical and mental, through functional recovery.

This study found that adult equinocavovarus foot patients with preoperative anxiety/depression symptoms exhibited significantly worse scores across all clinical outcome measures at baseline compared to those without such symptoms, suggesting a complex interplay between mental factors and clinical manifestations. Studies have indicated that anxiety and depression are not only associated with heightened pain perception but can also lead to aggravated functional limitations (15, 33–35). The interaction between mental factors and somatic symptoms involves complex neurobiological mechanisms. At the neurotransmitter level, research has revealed that pain modulation pathways share key neurotransmitters with emotion regulation systems, including serotonin and norepinephrine (36). Furthermore, neuroimaging studies have demonstrated that brain regions involved in pain pathways (e.g., the prefrontal cortex, hippocampus, and amygdala) overlap with those implicated in mood disorders (37, 38). We proposes that patients with preoperative psychological distress may experience stronger pain perception and reduce activity due to fear. This behavioral avoidance can exacerbate their pre-existing functional limitations, potentially creating a vicious cycle that further intensifies their level of psychological distress. As demonstrated in previous studies, pain and poor mental health are closely interrelated in a bidirectional manner (34). Specifically, an increase in pain intensity can predict the aggravation of negative emotions and conversely (33). Furthermore, depressive/anxious states have been confirmed to be closely related to poor functional recovery both in the early and late post-traumatic periods (39), and a higher level of preoperative anxiety/depression is correlated with greater preoperative pain intensity and lower quality of life (19).

At the final follow-up, patients with preoperative anxiety/depression symptoms continued to demonstrate significantly poorer outcomes across all clinical measures. This indicates that mental factors exert a substantial influence on the surgical management of adult equinocavovarus foot deformity. This finding is consistent with previous literature reports indicating that mental health status is an independent risk factor influencing prognosis in orthopedic surgery (18, 40, 41). The presence of anxiety/depression symptoms is independently associated with more severe pain and impaired quality of life (15). Pain may represent a somatic manifestation of underlying mental issues (36), which can prompt patients to avoid pain-inducing activities during postoperative rehabilitation, thereby perpetuating and promoting functional limitations (42, 43). We propose that during postoperative rehabilitation, patients form exaggerated negative assessments of their ability to engage in activities that may induce pain, leading to avoidance behavior, which results in physical deconditioning and exacerbation of pain during recovery (44, 45), which in turn adversely affects mental health. In recent years, within the field of foot and ankle surgery, multiple studies have consistently demonstrated that patients with higher levels of preoperative anxiety/depression exhibit significantly worse scores at baseline and during follow-up assessments across domains of pain, function, quality of life, and mental well-being, compared to patients with sound mental health (46–49). In a study of patients with hallux valgus, Goh et al. found that those with comorbid psychological distress not only exhibited poorer baseline scores on the VAS, AOFAS, and SF-36 (PCS/MCS) but also demonstrated significantly less improvement in pain, function, and mental metrics at the 6-month postoperative follow-up compared to the control group. Moreover, levels of psychological distress remained elevated even at the two-year postoperative mark (16). These findings provide a degree of corroborating evidence for the results of the present study.

This study further analyzed the magnitude of improvement in various clinical outcome measures from preoperative assessment to the final follow-up between the two groups. The results showed that the preoperative anxiety/depression group demonstrated significantly greater improvements in SF-36 (MCS), HADS-A, and HADS-D scores compared with the control group, but showed significantly less improvement in VAS, AOFAS, and SF-36 (PCS) scores. This differential pattern improvement suggests that preoperative psychological distress exerts a persistent negative influence on postoperative physical functional recovery. Notably, even after demonstrating significant mental health gains, these patients achieved a lesser degree of pain relief and functional restoration compared to their psychologically healthier counterparts. We postulate that patients with preoperative anxiety/depression symptoms had lower baseline mental scores, indicating greater room for improvement. This “ceiling effect” provides greater potential for postoperative gains in mental metrics. Furthermore, surgical correction of the deformity restores normal foot appearance and basic function, thereby addressing the primary etiology of the psychological distress and leading to further alleviation of the mental burden. This finding is consistent with results previously reported in patients with hallux valgus and painful accessory navicular syndrome (16, 32). However, The relative insufficiency in somatic symptoms and physical function improvement reflects the persistent impact of psychological factors on postoperative rehabilitation. which may be attributable to the amplifying effect of negative emotions on pain perception and their inhibitory effect on functional recovery. Cunningham et al. observed a analogous phenomenon in patients undergoing total ankle arthroplasty, where those with preoperative depression demonstrated significantly less improvement in both pain and SF-36 (PCS) scores compared to non-depressed patients (47). Separate research has indicated that, over the same rehabilitation period, patients without anxiety achieve functional recovery and pain relief at a significantly faster rate than those with anxiety (27).

This study not only investigated the clinical efficacy of corrective surgery for adult equinocavovarus foot but also explored the association between preoperative anxiety/depression symptoms and surgical outcomes, providing valuable reference for comprehensive treatment of this condition. However, this study has several limitations that should be acknowledged. Firstly, this was a retrospective observational study; therefore, we can only report associations between preoperative anxiety/depression and various indicators and cannot infer causality. Secondly, this was a single-center, retrospective study. All 83 patients were recruited from the same tertiary Grade A orthopedic specialty hospital. The relatively limited sample size may affect the generalizability of the findings. Thirdly, surgical procedures were not strictly standardized in this study. Although all surgeries were performed by the same senior surgical team following standardized principles, individualized adjustments may have introduced some heterogeneity. Fourthly, this study mainly focused on functional scores and mental health metrics, without incorporating radiographic anatomical parameters into the analysis. This omission may limit a comprehensive evaluation of the corrective surgical outcomes. Fifthly, HADS is a screening tool, not a diagnostic instrument; a score of ≥8 does not indicate a confirmed diagnosis of anxiety or depression. Sixthly, although standardized scales were adopted, the assessment of psychological symptoms remains susceptible to be influenced by patients’ subjective reporting and assessors’ interpretation, introducing a potential risk of information bias. Finally, the study did not fully incorporate other potentially significant confounding variables, such as patient’s socioeconomic status, educational level, rehabilitation compliance, and cultural background. These factors may affect treatment adherence and the quality of rehabilitation, thereby potentially confounding the association between mental status and surgical outcomes. Future research should consider conducting multi-center, large-sample prospective cohort studies or randomized controlled trials with extended follow-up periods, standardized surgical procedures, incorporating a more comprehensive set of outcome measures and control for potential confounding factors, in order to further elucidate the role of mental health status in the surgical management of equinocavovarus foot and provide higher-level evidence-based medical evidence for developing individualized and multidisciplinary comprehensive treatment strategies.

## Conclusion

Anxiety and depression symptoms are common negative mental states observed preoperatively in adult equinocavovarus foot patients and are associated with poor prognosis after corrective surgery. Although all patients achieved favorable physical and psychological outcomes following corrective surgery, those with preoperative anxiety/depression symptoms demonstrated significantly inferior surgical results. Notably, they exhibited a smaller magnitude of improvement in somatic symptoms and quality of life, yet a greater magnitude of improvement in mental health metrics. Clinicians should attach importance to the assessment of preoperative mental health status when formulating treatment plans. The implementation of necessary psychological interventions and tailored postoperative rehabilitation programs is essential to enhance overall surgical outcomes for these patients.

## Data Availability

The original contributions presented in the study are included in the article/supplementary material. Further inquiries can be directed to the corresponding author.
